# Otorrhœa

**Published:** 1889-02

**Authors:** 


### OTORRHŒA.

December, 1887.—The child of Mr. A. Skinner had diphtheria a month ago, which left her with running of the ears, which the attending physician said to let alone, as it was incurable, but she might outgrow it. There was an eczematous eruption behind the ears. The discharge was very fetid. She disliked to have her toilet made; mucous discharge from the nose.

Gave her two powders of Sulph.$^{cc}$, which produced a decided improvement for six weeks, then Sulph.$^{500}$, one powder, completed the cure, and she has continued well for nearly a year.

